# The Genetic and Endoplasmic Reticulum-Mediated Molecular Mechanisms of Primary Open-Angle Glaucoma

**DOI:** 10.3390/ijms21114171

**Published:** 2020-06-11

**Authors:** Wioletta Rozpędek-Kamińska, Radosław Wojtczak, Jacek P. Szaflik, Jerzy Szaflik, Ireneusz Majsterek

**Affiliations:** 1 Department of Clinical Chemistry and Biochemistry, Medical University of Lodz, 90-419 Lodz, Poland; wioletta.rozpedek@umed.lodz.pl (W.R.-K.); radoslaw.wojtczak@p.lodz.pl (R.W.); 2 Department of Ophthalmology, SPKSO Ophthalmic Hospital, Medical University of Warsaw, 03-709 Warsaw, Poland; jacek@szaflik.pl; 3 Laser Eye Microsurgery Center, Clinic of Jerzy Szaflik, 00-215 Warsaw, Poland; jerzy@szaflik.pl

**Keywords:** glaucoma, eye disease, hereditary, intraocular pressure, ocular hypertension, molecular pathologies, unfolded protein response, PERK, cell death

## Abstract

Glaucoma is a heterogenous, chronic, progressive group of eye diseases, which results in irreversible loss of vision. There are several types of glaucoma, whereas the primary open-angle glaucoma (POAG) constitutes the most common type of glaucoma, accounting for three-quarters of all glaucoma cases. The pathological mechanisms leading to POAG pathogenesis are multifactorial and still poorly understood, but it is commonly known that significantly elevated intraocular pressure (IOP) plays a crucial role in POAG pathogenesis. Besides, genetic predisposition and aggregation of abrogated proteins within the endoplasmic reticulum (ER) lumen and subsequent activation of the protein kinase RNA-like endoplasmic reticulum kinase (PERK)-dependent unfolded protein response (UPR) signaling pathway may also constitute important factors for POAG pathogenesis at the molecular level. Glaucoma is commonly known as a ‘silent thief of sight’, as it remains asymptomatic until later stages, and thus its diagnosis is frequently delayed. Thereby, detailed knowledge about the glaucoma pathophysiology is necessary to develop both biochemical and genetic tests to improve its early diagnosis as well as develop a novel, ground-breaking treatment strategy, as currently used medical therapies against glaucoma are limited and may evoke numerous adverse side-effects in patients.

## 1. Introduction

Glaucoma is a chronic and progressive disease affecting the structures of the eye, leading to the optic nerve atrophy, to apoptosis of retinal ganglion cells (RGCs), and finally to loss of vision [1,2,3]. It has also been reported that a common feature of glaucoma is a thinning of the retinal nerve fibre layer as well as cupping of the optic disc [4,5,6,7]. According to the morphology of the anterior chamber angle, glaucoma may by subdivided into open-angle glaucoma (OAG) and angle-closure glaucoma (ACG) [8,9]. The intra-ocular pressure (IOP) is determined by the balance between secretion of aqueous humor by the ciliary body and its drainage through both the trabecular meshwork (TM) and uveoscleral outflow pathway. Increased resistance to aqueous outflow via the TM is a characteristic of individuals with OAG, whereas in individuals with ACG, access to the drainage pathway is obstructed [10]. Both OAG and ACG may constitute a primary disease [10], whereas a secondary glaucoma may develop inter alia as a result of trauma [11], intake of medications like corticosteroids [12,13], inflammation [14], or specific conditions such as pigment dispersion or pseudo-exfoliation [15,16,17].

Despite many studies, the precise etiology of the glaucoma has not yet been determined, and factors that may play a crucial role in the disease progression have not been characterized in detail [18]. It is commonly known that glaucoma development and progression are strictly correlated with the pathophysiology of the optic nerve, the rate and severity of which is affected by the level of IOP. Interestingly, the level of IOP may determine whether or not the etiologic factors will evoke glaucomatous damage. It has been demonstrated that, in approximately half the people with IOP of 35 mmHg or higher, the glaucoma and field loss have been diagnosed [19], whereas a lower percentage of individuals have developed glaucoma over several years with the IOP ranging from 21 to 30 mmHg. Thus, the elevated level of IOP is associated with most forms of glaucoma, and is currently the only known modifiable risk factor for glaucoma [20].

Glaucoma has relatively high prevalence, as it has been reported that it constitutes the second cause of global blindness, after cataract. Cataract accounts for 47.8% of blindness worldwide, whereas glaucoma accounts for 12.3% of blindness worldwide. It has also been demonstrated that glaucoma-associated visual impairment is more severe in the least developed regions, and affects adults more often than children, as well as women more than men [21]. The number of people affected by the glaucoma is still increasing and it is estimated that, in 2040, it may reach up to 111.8 million worldwide [22]. It has been reported that 79.6 million people worldwide will be diagnosed with OAG and ACG by 2020, and 5.9 million individuals with OAG and 5.3 million individuals with ACG will be bilaterally blind [23]. Glaucoma is commonly called a ‘silent thief of sight’, as it may remain asymptomatic at the relatively late stage. It has been demonstrated that there is a high frequency of undiagnosed glaucoma cases worldwide, thereby many individuals that suffer from glaucoma are unaware about the disease progression [24,25,26,27,28].

## 2. Primary Open-Angle Glaucoma and Primary Angle-Closure Glaucoma

**Figure 1:**
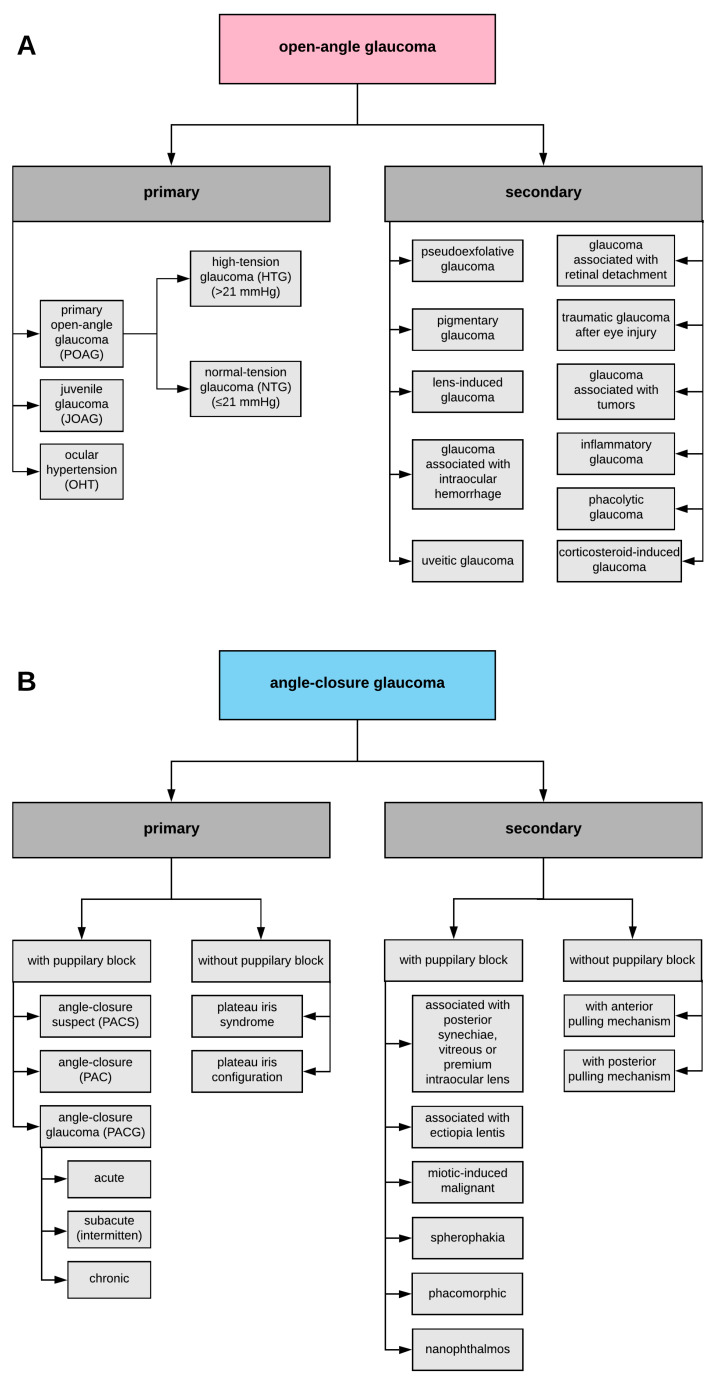
Clinical classification of open-angle glaucoma (OAG) (**A**) and angle-closure glaucoma (ACG) (**B**).

**Figure 2:**
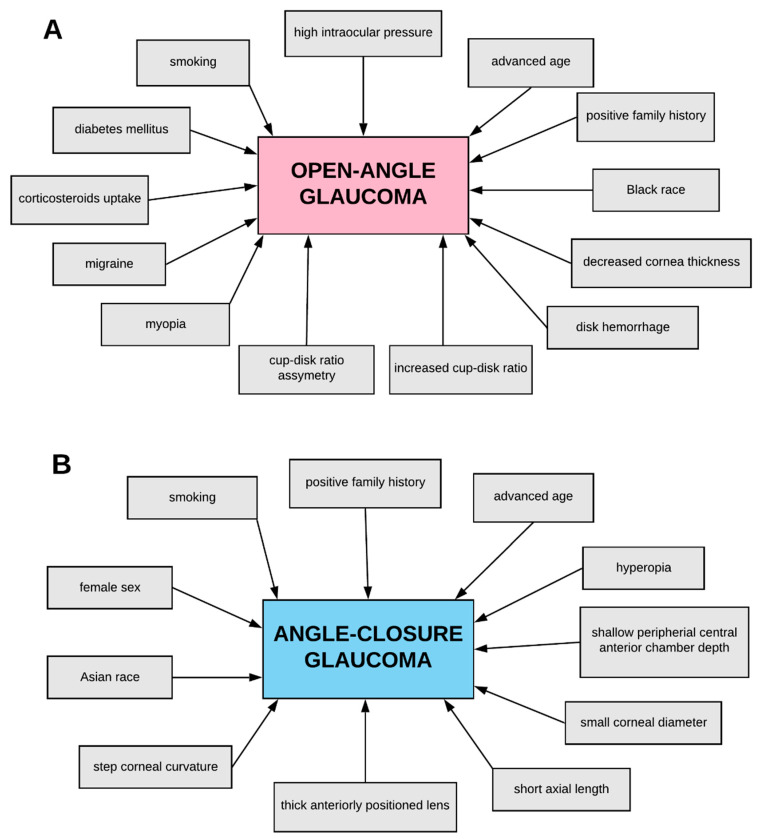
Risk factors for open-angle glaucoma (OAG) (**A**) and angle-closure glaucoma (ACG) (**B**).

There are many subtypes of glaucoma (Figure 1), whereas primary open-angle glaucoma (POAG) constitutes the most common type of glaucoma [29]. Adult-onset POAG affects individuals after 40 years of age [30], whereas early-onset POAG (juvenile POAG) affects younger individuals between 3 years of age and early adulthood [31]. It has been demonstrated that POAG is an autosomal dominant disease and its common clinical feature constitutes an elevated level of IOP [32,33]. POAG is clinically characterized by an open iridocorneal angle; damage of the optic nerve, including optic disc cupping; loss of RGCs; and, finally, defects in the visual field [20,34]. Risk factors for POAG include, among others, high IOP, positive family history, advanced age, black race, increased cup–disk ratio (CDR), CDR asymmetry, and disc hemorrhage, as well as corticosteroids intake (Figure 2A) [35,36,37]. Among the above-mentioned factors, an elevated level of IOP constitutes the most common cause of POAG development. As the IOP patients are categorized into high tension glaucoma (HTG) or normal tension glaucoma (NTG) subgroups [38], the NTG constitutes one of the POAG subtypes, comprising a special form of glaucomatous neurodegeneration or glaucomatous optic neuropathy (GON) almost exactly the same as that in POAG, whereas the IOP remains in the normal range, thus it equals 21 mmHg or less [39,40,41]. Most NTG individuals suffer from adult-onset disease, however, the disease may sporadically have early onset with autosomal dominant inheritance [42,43,44]. The etiology of NTG is multifactorial and still not fully elucidated. Multiple structural and functional differences provide clear evidence that various mechanisms may be strictly correlated with the pathogenesis of NTG. It has been reported that common risk factors for the development and progression of NTG may be associated with general status as low blood pressure [45], migraines [46], dysregulation of blood flow [47], and diabetes mellitus [48], as well as optic disc hemorrhages [49] and parapapillary atrophy [50]. Interestingly, it has been demonstrated that central corneal thickness (CCT) constitutes a crucial clinical factor to precisely determine glaucoma severity during the initial examination. Evaluation of CCT may be useful for identification of glaucoma individuals at high risk for disease progression. Lower CCT was closely correlated with worse both Advanced Glaucoma Intervention Study score and mean deviation of visual field, as well as with increased vertical and horizontal cup–disc ratios (CDRs) [51]. The CCT constitutes a crucial factor that should be measured to precisely interpret the IOP measurements [52]. Shih et al. have reported that measurement of CCT has an important impact both on the clinical management of individuals with diagnosed glaucoma and glaucoma suspects [53]. Study by Shetgar and Mulimani has demonstrated that CCT was markedly lower in NTG glaucoma patients as compared with control and POAG patients. However, the ocular hypertension (OHT) patients have been characterized by significantly higher CCT as compared with controls and POAG patients. Owing to the significant impact of CCT on IOP measurement, which constitutes not only a major glaucoma diagnostic parameter, but also an important parameter to follow up a disease progression, a significant number of glaucoma patients are misdiagnosed to improper glaucoma subtypes. Thereby, evaluation of the CCT is key to making a correct diagnosis and to management of glaucoma individuals and glaucoma suspects [54]. The above-mentioned data have been consistent with the results obtained by René-Pierre et al., which also demonstrated that NTG patients have been characterized by lower CCT in comparison with control group and POAG individuals. Moreover, in the mentioned study, in OHT patients, higher CCT has been diagnosed as compared with the control group and POAG patients [55]. Moreover, Doyle et al. have reported that CCT was significantly lower in NTG individuals as compared with POAG individuals. Furthermore, lower CCT was noted in NTG patients with vascular risk factors as compared with patients without vascular risk factors [56]. It has also been demonstrated that glaucoma individuals with thin CCT were more often at an advanced stage of the disease and also represented NTG patients and black African ancestry [57]. Furthermore, a study by Henderson et al. has shown that OHT individuals with thinner CCT have been characterized by markedly lower retinal nerve fibre layer thickness measurements as compared with control subjects and OHT individuals with thicker CCT. Thereby, mentioned research has suggested that different CCT measurements may be connected with different possibilities for glaucoma damage development [58]. It has been reported that scleral thickness and CCT are characterized by a moderate positive correlation. Stress plays a key role in glaucoma pathogenesis and evokes retinal layers malformations as well as, finally, significant neuronal tissue strain. Progression of glaucoma damage is inversely correlated with the CCT. When the CCT decreases, the level of stress increases inversely, which consequently evokes significant interruption of retinal layers and higher levels of neural tissue strain, which increases the risk of glaucoma development and progression. The above-mentioned hypothesis may constitute an explanation of the association of low CCT and increased susceptibility to glaucoma damage in NTG patients [59].

As mentioned above, there are multiple risk factors that contribute to glaucoma development and progression, whereas it has also been reported that autoimmune mechanisms may play a crucial role in glaucoma pathogenesis. Thereby, detailed knowledge concerning the role of the immune system in glaucoma development and progression may contribute to better understanding of the disease pathogenesis as well as to the development of a novel treatment strategy against glaucoma [60]. It has been reported that immunoregulation plays a central role in determination of whether RGCs survive or undergo apoptotic cell death in glaucoma patients [61]. Wax et al. have demonstrated an increased antibody reactivity in NTG individuals. Thereby, it has been reported that immune mechanisms may play an important role in the pathogenesis of optic neuropathy in NTG patients [62,63]. Tezel et al. have demonstrated an elevated level of antibodies against small heat shock proteins (HSPs) such as alpha-crystallins and HSP27 in NTG individuals. Furthermore, NTG individuals have been characterized by a higher titer of autoantibodies to small HSPs as compared with control subjects or individuals with high-pressure glaucoma. Interestingly, antibodies against small HSPs had a pathogenic significance in glaucoma patients, as being applied to retina tissue or cells evoked their apoptotic cell death [64]. Elevated levels of both HSP27 and HSP60 have been demonstrated in the human donor glaucomatous eyes as compared with normal eyes from age-matched donors [65]. Grus et al. have shown complex antibody profiles against optic nerve antigens in sera of glaucoma patients (POAG, NTG, and OHT) and control subjects. The mentioned analysis indicated that several molecular weight regions, characterized by an increased antibody reactivity, were present, especially in the NTG patients. Moreover, several regions with lower reactivities have been found in the NTG individuals as compared with other analysed groups. Besides, Grus et al. evaluated the IgG autoantibody repertoires in sera of glaucoma individuals against optic nerve antigens. They demonstrated a significant difference between all analyzed groups against optic nerve antigens. Interestingly, the NTG individuals have been characterized by the highest variance from controls (*p* < 0.01). The above-mentioned research has shown immunological effects in both POAG and NTG patients, and has suggested that autoantibodies may play an important role in both NTG and POAG pathogenesis [66]. It has also been revealed that serum autoantibodies to α-fodrin, also typical of other neurodegenerative disease, have been present in glaucoma individuals from German and the United States. Thereby, it has been suggested that an α-fodrin may constitute antibody biomarker in both study populations. The above-mentioned analysis has shown an increased frequency and immunoreactivity to α-fodrin, especially in the sera of NTG patients. The results obtained in this study suggested a significant role of autoimmunity and the neurodegenerative processes in glaucoma pathogenesis [67]. A study by Gramlich et al. has demonstrated that IgG antibodies and plasma cells are deposed in human glaucomatous retina. Furthermore, deposits of IgG have been found in a pro-inflammatory environment, with accompanying increased levels of TNF-a, IL-6, and IL-8, which may be maintained locally by immune-competent cells such as microglia. The above-mentioned research has indicated an immunological involvement in glaucoma, like in the pathogenesis of other multiple neurodegenerative diseases, and it presents pathogenic mechanisms, which are closely correlated with the unique nature of the eye and retina [68].

Primary angle-closure glaucoma (PACG) also constitutes a common cause of blindness, as it is said to be responsible for nearly half of the cases of glaucoma-related blindness worldwide [69,70]. PACG, as compared with POAG, is characterized by an anatomically closed angle. ACG typically results from abnormal anatomy of the anterior segment of the eye, such as a narrow anterior chamber angle, a shallow anterior chamber depth, a thicker lens, a more anterior lens position, a small corneal diameter, or a shorter axial length [71,72,73,74]. ACG is caused by uveal effusion and anterior rotation of the ciliary body with resultant closure of the iridocorneal angle [75]. Pupillary block constitutes the most common mechanism of an angle closure and is evoked by the resistance of aqueous humor to flow from the posterior towards anterior chambers through the pupil. Aqueous humor accumulates behind the iris, which increases its convexity and finally leads to angle closure [76,77,78]. PACG is commonly classified into primary angle-closure suspect (PACS), primary angle closure (PAC), and PACG itself [79,80]. There are numerous risk factors leading to PACG development, whereas it has been demonstrated that the prevalence of PACG development is higher primarily among women, the elderly, and hyperopic individuals, and it is most prevalent in Asian ethnicity (Figure 2B) [23,81,82,83,84,85].

## 3. Genes as Risk Factors for POAG Pathogenesis

There is ample evidence that genes play a crucial role in the pathogenesis of multiple eye diseases, including POAG. Detailed research of the disease-inducing genes provides important data closely connected with the pathogenesis of heritable eye disease, as the disease-causing genes may constitute a part of a key biological signaling pathways that, after detailed investigation, may explain the molecular mechanisms responsible for the diseases pathogenesis and progression. Moreover, identification of the disease-inducing genes may contribute to the development of the DNA-based tests useful for the assessment of patient’s risk for the disease and to distinguish between clinically similar disorders. Identification of the specific mutations may be important for the prediction of the clinical course of the disease [86]. The prevention and early diagnosis of glaucoma require the evaluation of various genetic and environmental risk factors as well as IOP [87]. Currently, glaucoma therapies are limited, as they are primarily based on the reduction of the elevated IOP, as a major risk factor for POAG development [88]. Although IOP has a huge influence on the glaucoma development, genetic factors also have a considerable impact on the pathomechanism of glaucoma [89]. Multiple studies in the recent decades have identified numerous genes and genetic risk factors that play a key role in glaucoma pathogenesis. The above-mentioned investigations significantly increased knowledge about the disease mechanisms, which is important for the development of new diagnostic tools and novel therapies against glaucoma [90].

Early-onset glaucoma may affect children and young adults and it is predominantly inherited as Mendelian autosomal dominant or recessive traits, whereas glaucoma affecting older individuals is characterized by a complex inheritance [88]. Genetic mutations responsible for early-onset glaucoma development are rare and are characterized by large biological impact, and thus high penetrance. Variants of genes contributing to adult-onset glaucoma are common and have a small, incremental effect on the disease development and only combined effects of multiple risk factors, including environmental risk factors, may evoke a significantly larger impact on the disease pathogenesis [86,88,91]. It has been reported that there are at least 20 genomic regions strictly correlated with POAG pathogenesis [92]. Variants of genes with rare frequency and high effect size, which lead to the development of POAG include *myocilin* (*MYOC*), *WD repeat domain 36* (*WDR36*), *optineurin* (*OPTN*), *TANK-binding kinase 1* (*TBK1*), as well as *neurotrophin 4* (*NTF4*) [42,88,93,94,95,96,97,98]. Moreover, mutations in *paired box 6* (*PAX6*) gene are rare with large biological effect and are closely associated with the pathogenesis of developmental glaucoma related to anterior segment dysgenesis [91]. Variants of genes with common frequency and low effect size leading to the development of POAG include the following: *Cyclin-dependent kinase inhibitor 2B* (*CDKN2BAS*), *caveolin 1* and *caveolin 2 (CAV1/CAV2), sine oculis homeobox homolog 1* and *sine oculis homeobox homolog 6 (SIX1/SIX6), transmembrane and coiled-coil domains 1 (TMCO1), growth arrest specific 7 (GAS7), atonal homolog 7 (ATOH7)*, and *RPGR Interacting Protein 1 (RPGRIP1)* [88,91,99,100,101,102,103,104,105,106,107].

### 3.1. Rare Variants of Genes with High Effect Size Correlated with POAG Pathogenesis

#### 3.1.1. *MYOC*

*MYOC,* which encodes myocilin protein, constitutes the first identified gene linked to POAG pathogenesis. It has been found at locus GLC1A on chromosome 1q23-25 [108]. As a consequence of its independent discovery by several laboratories *MYOC* is also known as a *trabecular meshwork inducible glucocorticoid response* (*TIGR*), *GLC1A*, *myocilin,* or *TIGR/myocilin* gene [109,110]. MYOC protein is mainly present in the ocular tissue in the TM cells, the Schlemm’s canal, the sclera, the ciliary body, the retina, as well as the optic nerve [111,112]. It has been reported that mutant myocilin is poorly secreted and aggregated within TM cells. Accumulated abnormal myocilin protein may be toxic towards TM cells and may subsequently evoke their dysfunction or apoptotic cell death, which may eventually result in decreased aqueous outflow, elevated IOP, and subsequent glaucoma development [40,113,114,115,116,117,118]. Interestingly, research by Kasetti et al. has demonstrated that mutant myocilin directly triggers abnormal accumulation of the extracellular matrix in the endoplasmic reticulum (ER) of TM cells, which may decrease aqueous humor outflow facility as well as evoke IOP elevation in myocilin-associated glaucoma [119].

Polansky et al. have demonstrated induction of the expression of a 57kD myocilin protein in human TM cells treated with dexamethasone [120]. Mutations in *MYOC* have been identified in 2–4% of individuals suffering from POAG worldwide [41]. It has been demonstrated that *MYOC* mutations are responsible for most cases of autosomal dominant juvenile-onset POAG, and they cause up to 4.6% of cases of adult-onset POAG. The prevalence of *MYOC* mutations is similar regardless of race or geographic location [40,121]. The most commonly identified *MYOC* mutation constitutes Gln368Stop and it has been identified in POAG individuals of all racial groups, with the highest frequency among Caucasian subjects. A few instances of the Gln368Stop mutation have been reported in African American and Indian POAG individuals [40,121,122]. It has been demonstrated that MYOC^Q368X^ constitutes the most frequent variation responsible for late-onset POAG development, with an average age of 59 years at the date of diagnosis, whereas the Y437H mutation is responsible for early-onset glaucoma with an average age of onset of 20 years [115]. Besides, it has been reported that C1456T mutation in *MYOC* was responsible for the POAG pathogenesis in the Chinese family [123].

#### 3.1.2. *WDR36*

Another gene correlated with glaucoma pathogenesis constitutes *WDR36*, which contains several iterations of the WD40 repeat motif (*WD40-repeat 36*). *WDR36* expression has been reported both in multiple non-ocular and ocular tissues including lens, iris, sclera, ciliary muscles, ciliary body, TM, retina, and optic nerve. *WRD36* encodes protein, the function of which still remains poorly understood, whereas it has been predicted that *WRD36* may be a causative gene for the adult-onset POAG development at the GLC1G locus. It has been suggested that the pathoetiology of both high- and low-pressure glaucoma may be correlated with *WDR36* specific expression in ocular tissues as well as with mutations present in the *WDR36* gene [124]. Interestingly, it has been demonstrated that abnormalities in *WDR36* alone are not sufficient for POAG development, whereas correlation of *WDR36* sequence variants with more severe disease in POAG patients suggests that abnormalities in the *WDR36* may lead to POAG development, and also that *WDR36* may constitute glaucoma modifier gene [125]. Investigation by Skarie et al. has demonstrated that *Wdr36* in zebrafish, a homolog of human *WDR36*, constitutes a functional homolog of the *Utp21* in yeast, which is a component of the rRNA processome, and it is directly involved in 18S rRNA processing and nucleolar homeostasis. Furthermore, *Wdr36* loss of function evokes ocular dysmorphology and activation of the p53 stress-response signaling pathway. Thereby, *WRD36* may play a causative or modifying roles in the POAG pathology [126]. Moreover, it has been demonstrated that five *Utp21p* variants, homologous to L25P, R529Q, I604V, D658G, and M671V in human *WDR36*, resulted in growth defects with significant changes in the pre-rRNA levels. Thus, non-synonymous amino acid variations in *WDR36* alter protein function and evoke deleterious cellular conditions, which may be directly correlated with POAG pathogenesis [127]. A study by Chi et al. has revealed that *WDR36* plays a crucial role in the retina homeostasis and *WDR36* mutation may be responsible for the progressive devastating retinal damage [128]. Interestingly, it has been suggested that *WDR36* may constitute a minor disease-causing gene in POAG in the German population [94], whereas in Chinese individuals, *WDR36* may be correlated only with sporadic HTG, but not with NTG or JOAG. Additionally, Fan et al. have suggested a different *WDR36* mutation pattern in the Chinese population from other ethnic populations [129].

#### 3.1.3. *OPTN*

*OPTN*, an adaptor protein, is directly involved in mediation of variety of cellular processes including cell signaling, vesicle trafficking, and autophagy [130,131,132]. *OPTN* is expressed in multiple human ocular tissues including TM, cornea, nonpigmented ciliary epithelium, iris, and retina [133,134]. Moreover, *OPTN* has also been found in the aqueous humour, thus it may be classified as a secretory protein [42]. To explain in detail the glaucoma pathogenesis, a cytoprotective role of *OPTN* has been proposed. It has been reported that *OPTN* plays a crucial role in the neurotrophins secretion, which is necessary for cell survival [135].

Some *OPTN* mutations are correlated with POAG pathogenesis, whereas glaucoma-associated *OPTN* mutations constitute mostly missense mutations [136,137]. *OPTN* mutations have been reported in 16.7% of families with hereditary POAG, whereas most of them have been associated with NTG [42]. Glaucoma-associated missense mutations of *OPTN* include, among others, E50K [138], H26D, H486R, and E322K, whereas E50K constitutes the most common *OPTN* mutation and is strictly associated with the more severe form of glaucoma [138,139,140,141,142,143]. Furthermore, it has been demonstrated that *OPTN* mutation is correlated with accumulation of damaged mitochondria and disrupted mitophagy. Shim et al. have reported that *OPTN* E50K mutation is closely connected with activation of oxidative stress and apoptotic signaling pathway and triggers dynamics alteration-mediated mitochondrial degradation in RGCs. Moreover, expression of E50K *OPTN* triggered mitochondrial fission-mediated mitochondrial degradation and mitophagy in the glial lamina of aged E50K^−tg^ mice [144]. The transgenic mice with overexpression of E50K *OPTN* demonstrated diffused retinal layers with thinner retina in comparison with the mice with low expression of E50K *OPTN* [145]. Individuals with glaucoma and the *OPTN* E50K mutation have been found to have NTG that was more severe than that in a control group of individuals with NTG without the *OPTN* E50K mutation [138]. Besides, E50K mutant mice exhibited histological abnormalities in the retina, massive apoptosis, and degeneration of entire retina resulting in approximately a 28% reduction of the retina thickness. It has also been demonstrated that *OPTN* E50K mutation-mediated glaucoma may be triggered via disruption of interaction between *OPTN* and Rab8 GTPase [146].

Interestingly, it has been reported that POAG individuals with Glu50Lys mutation in *OPTN* have primarily exhibited early-onset of severe optic nerve damage that occurs without IOP elevation [147]. Two *OPTN* mutations, Glu50Lys and Arg545Gln, have been identified in several studies of NTG patients, whereas data confirming the Glu50Lys mutation with NTG pathogenesis are stronger [42,148,149]. It has been reported that NTG patients with the Gln50Lys mutation exhibited a lower level of IOP, larger CDR, more visual field loss, as well as higher rate of surgery than NTG subjects without Gln50Lys mutation [138]. Furthermore, another variant of the *OPTN* gene, Met98Lys, has been detected more frequently in NTG patients, primarily in Asian cohorts [142,148,150].

#### 3.1.4. *TBK1*

*TBK1*, an IκB kinase (IKK)-related kinase, is associated with interferon regulatory factor (IRF)- and nuclear factor (NF)-κB-activation [151]. Thereby, it is correlated with innate immune defense and its dysregulation may have a significant impact on pathogenesis of multiple diseases [152,153,154]. *TBK1*, in order to promote an innate immunity by modulating transcription, may activate autophagy proteins *OPTN* and p62 [155,156]. *TBK1* also plays a key role in clearance of intracellular protein aggregates and damaged organelles [157,158].

Duplication of the *TBK1* gene is directly correlated with 1–2% cases of NTG [159]. In in vitro research by Trucker et al., a cellular model of RGC-like neurons differentiated from skin-derived induced pluripotent stem cells from *TBK1*-associated NTG individuals, as well as from normal control subjects, has been used. It was demonstrated that both fibroblasts and RGC-like neurons derived from NTG patients with *TBK1* gene duplication exhibited significantly increased level of one of the key markers of autophagy, LC3-II protein. Hence, the above-mentioned study has suggested that dysregulation of this catabolic pathway may result in *TBK1*-associated glaucoma development [160]. Fingert et al., in in vivo experimental model, have confirmed the pathogenicity of the *TBK1* gene duplication in human NTG and suggested that overexpression of *TBK1* may play an important role in glaucoma pathology. In the mentioned study, transgenic mice with a copy of the human *TBK1* (Tg-*TBK1*) were used. It was demonstrated that *TBK1* were primarily localized within ganglion cell layer of the retina. A higher concentration of the *TBK1* labelling was exhibited in RGCs of g-*TBK1* mice, as compared with wild-type mice. Besides, in Tg-*TBK1* mice, the loss of RGCs was confirmed to be progressive. Tg-*TBK1* mice with higher doses of the *TBK1* gene exhibited the phenotype of human *TBK1*-associated NTG [159]. In another study, it has been demonstrated that *TBK1* is expressed in ganglion cells and the retinal nerve fiber layer [161]. Research by Fingert et al. links the duplication of genes located on chromosome 12q14, including *TBK1*, with familial NTG and suggested that an extra copy of the *TBK1* gene is responsible for NTG pathogenesis [162]. Furthermore, Morton et al. have suggested that protein encoded by *OPTN*, a gene also associated with NTG, may directly interact with *TBK1*, which supports its role in glaucoma pathogenesis. The mutant E50K *OPTN* correlated with POAG displayed strikingly enhanced binding to *TBK1*, which may contribute to the familial POAG caused by this mutation [43].

#### 3.1.5. *NTF4*

Human *NTF4* gene is located on chromosome 19q13.33, which was previously identified as a putative glaucoma locus in a genome-wide linkage scan [163,164]. *NTF4* belongs to the neurotrophin protein family. It has been reported that *NTF4* plays a key role in the activation of tyrosine kinase B (TrkB) receptor on RGCs and prevents their apoptotic cell death in in vitro cellular models as well as in in vivo animal models after axotomy [163,165,166,167,168,169]. NFT4 is also involved in the postnatal survival of retinal neurons during development and degeneration [170]. In the literature data, the role of *NTF4* in POAG pathogenesis remains controversial, whereas *NTF4* has not been identified as a POAG-causing gene in several studies [171,172]. However, Pasutto et al. have reported seven different heterozygous *NTF4* mutations accounting for about 1.7% of POAG European individuals [97]. Interestingly, Vithana et al. have suggested that *NTF4* disease-causing mutations may be ethnic specific, because, in the Chinese cohort, they did not identify any of the *NTF4* mutations previously reported in European POAG individuals, including the most frequent mutation R206W. Furthermore, their findings of only a single, novel Leu113Ser mutation indicate that *NTF4* mutations are a rare cause of POAG in the Chinese individuals [98].

#### 3.1.6. *PAX6*

*PAX6* gene is located on 11p13 region on chromosome 11 [173]. *PAX6* protein belongs to the paired box family of transcription factors. It has been reported that *PAX6* is active in epithelial and mesenchymal cells during ocular development and plays a crucial role in synchronization of the complex interaction of cell types of different origin, which are responsible for proper morphogenesis of the anterior eye [174]. Moreover, *PAX6* plays an important role in maintaining the multipotent state of progenitor cells, such as neuronal retina, pigment epithelium of retina, iris, ciliary body, and cortex, as well as some subcortical brain structures, and their proliferation [175]. It has been demonstrated on the zebrafish model of corneal disease that *PAX6b* mutants embryos have been characterized by a thick cornea, iris hypoplasia, a shallow anterior chamber, as well as a small lens. Besides, ultrastructure analysis has shown a disrupted corneal endothelium. Interestingly, *PAX6b* mutants have demonstrated loss of corneal epithelial expression of genes, also including regulatory genes. Loss of *PAX6b* function also results in significant changes in the gene regulation program [176].

It has been demonstrated that occurrence of *PAX6* mutations may result in the development of aniridia, which constitutes a severe panocular eye disease associated with iris hypoplasia [177,178,179,180]. There are several research data confirming a direct correlation of *PAX6* gene mutation with aniridia occurrence [181,182,183,184]. Interestingly, it has been reported that aniridia is frequently correlated with glaucoma and glaucoma associated with aniridia may trigger a progressive loss of vision [185]. Netland et al. have reported that, in 46% individuals, out of 83 aniridia subjects, glaucoma has been identified [186]. Furthermore, Mayer et al. have shown that glaucoma has been identified in 52% out of 80 patients with congenital aniridia [187]. Lin et al. have demonstrated that aniridia associated with glaucoma, congenital cataract, and lens subluxation may be caused by the recurrent nonsense mutation c.718C > T (p.Arg240X) in exon 9 of the *PAX6* gene [188]. It has also been demonstrated that loss of *PAX6* expression may cause an aniridia occurrence [189,190,191]. Owing to the direct correlation of aniridia and glaucoma occurrence, Liu et al. have demonstrated that *PAX6* expression has also been markedly downregulated in non-myocilin POAG cases as compared with controls [192]. Research by Kroeber et al. has shown that somatic inactivation of one allele of *PAX6* from the epithelial cells of lens and cornea disrupted development of both TM and Schlemm’s canal. Furthermore, it also results in a growing adhesion between iris periphery and cornea in juvenile eyes, which triggers a complete closure of the iridocorneal angle in the adult eye. The above-mentioned structural malformations evoke a significant increase of the IOP and, consequently, optic nerve axon degeneration and glaucoma development [193].

### 3.2. Common Variants of Genes with Modest Effect Size Correlated with POAG Pathogenesis

#### 3.2.1. *CDKN2BAS*

*CDKN2BAS*, also known as an *antisense non-coding RNA in the INK4 locus* (*ANRIL)*, is located on chromosome 9p21. It has been reported that *CDKN2BAS* is directly correlated with the pathogenesis of multiple human diseases including type 2 diabetes, endometriosis, intracranial aneurysma, megakaryopoiesis, coronary artery disease, and periodontitis, as well as with several forms of cancer such as prostate cancer, stomach cancer, pancreatic cancer, leukemia, glioma, colorectal cancer, and lung cancer [194,195,196,197,198,199]. The role of *CDKN2BAS* still remains not fully understood, whereas it has been reported that *CDKN2BAS* is involved in regulation of the expression of *CDKN2A* and *CDKN2B* coding cyclin-dependent kinase inhibitors. CDKN2A and CDKN2B play a crucial role in cellular proliferation and block cell cycle progression, and have an important influence on physiological processes including replicative senescence, apoptosis, as well as stem-cell self-renewal [200]. There is ample evidence that occurrence of *CDKN2BAS* polymorphisms may contribute to the alteration in the expression of target genes, which play a key role in cell cycle regulation and may contribute to the RGCs’ apoptosis, and subsequently to glaucoma development [201].

Burdon et al. have demonstrated a strong association of *CDKN2BAS* with advanced OAG. Additionally, a retinal expression of *CDKN2BAS* in human ocular tissues has also been reported. CDKN2A and CDKN2B were significantly upregulated in the retina of a rat model of glaucoma [100]. Pasquale et al. have reported that alleles of *CDKN2BAS1* single nucleotide polymorphisms, which influence the risk of developing POAG, may also have a significant impact on optic nerve degeneration among POAG individuals, which indicates an important role of CDKN2BAS1 in POAG pathogenesis [202]. It has been demonstrated by Cao et al. that single nucleotide polymorphism rs1063192, located near the *CDKN2B*, is associated with POAG, and the minor allele C of rs1063192 is protective against POAG in the Afro-Caribbean population of Barbados. Research by Cao et al. has suggested that rs1063912 constitutes a common protective variant for POAG in both African and European descent [203]. Restrepo et al. have suggested that *CDKN2BAS1* constitutes a crucial locus for POAG risk among African Americans, as they have reported a direct correlation between the risk of POAG and African genetic ancestry at *CDKN2BAS1* [204].

The optic nerve head is involved in many ophthalmic disorders including POAG. Two of the most important parameters such as the size of the optic disc area and the vertical cup–disc ratio (VCDR) are highly heritable. A study by Ramdas et al. has shown that single-nucleotide polymorphism rs1063192 in *CDKN2BAS* on chromosome 9p21 is associated with VCDR, and thereby with POAG pathogenesis. Moreover, it has been reported that *CDKN2B* is implicated in transforming growth factor beta (TGFβ) signaling pathway [205]. It has been reported that the characteristic cupping of the optic nerve head in glaucoma is strictly correlated with TGFβ as well as with elevated biosynthesis and deposition of extracellular matrix (ECM) proteins [206]. Kasetti et al. have demonstrated that glucocorticoid such as dexamethasone triggers activation of TGFβ signaling pathway, which results in increased ECM accumulation and ER stress activation in the TM as well as significant elevation of IOP. Besides, dexamethasone induced TGFβ2 in the aqueous humor and TM of a mouse model of OHT. Hence, the above-mentioned results suggested that targeting of the TGFβ signaling pathway may constitute a promising therapy against glaucoma [207].

#### 3.2.2. *CAV1/CAV2*

*CAV1* and *CAV2* code for caveolin 1 and caveolin 2, respectively, both of which belong to the caveolin family proteins [208,209]. Caveolins play a crucial role in multiple cellular processes such as vesicular transport, cholesterol homeostasis, and signal transduction [210,211,212,213]. It has been demonstrated that they are expressed in ocular tissues such as human retina, ciliary muscle, TM, and Schlemm’s canal [214]. Caveolins inhibit endothelial nitric oxide synthase activity in the caveolae, which may evoke significant changes in vascular tone and TM function, which are closely associated with POAG pathogenesis [215,216,217,218,219] It has also been reported that caveolin 1 plays a major role in the IOP maintenance via modulation of aqueous humor drainage from the eye. It has been demonstrated in vivo that *CAV1*-deficient mice exhibited OHT via aberrant pressure-dependent drainage of aqueous humor. Deficiency of *CAV1* induces loss of caveolae in both the Schlemm’s canal and TM. Besides, an aqueous drainage from *CAV1*-deficient eyes was more sensitive to nitric oxide synthase inhibition than in used controls. Thereby, the mentioned results indicate a direct link between a glaucoma risk gene and glaucoma pathology [220].

Genome-wide association studies (GWAS) on an Icelandic cohort showed that variant rs4236601 in *CAV1* and *CAV2* on chromosome 7q31 has a significant influence on POAG pathogenesis, whereas it has been suggested that the mentioned correlation is dependent on the population [214]. Thorleifsson et al. have identified a variant rs4236601 and demonstrated that it is strictly correlated with POAG pathogenesis in European and east Asian individuals. It has been shown that rs4236601 has no impact on other common POAG risk factors including increased IOP level and CCT, as well as type 2 diabetes, hypertension, and myopia. It has been demonstrated that mentioned variant is located close to the *CAV1* and *CAV2*, both of which are expressed in the TM and RGCs. Thorleifsson et al. have shown that frequency of the rs4236601 variant is lower in east Asian individuals than in individuals of European ancestry [102]. Additional research by Nunes et al. has also confirmed that variant rs4236601 is correlated with POAG pathogenesis, as it has been demonstrated that it contributes to the incidence of POAG in a sample of the Brazilian Southeastern population [221]. Research by Rong et al. has confirmed the association of rs4236601 with POAG in the southern and northern Chinese HTG patients, and also identified a common single nucleotide polymorphism rs3801994 at the *CAV1/CAV2* locus in Chinese and Japanese individuals [222]. A recent study by Lu et al. has confirmed a correlation between rs4236601 at the *CAV1/CAV2* locus and NTG pathogenesis in Chinese individuals [223]. Furthermore, Loomis et al. have suggested a direct association between *CAV1/CAV2* single nucleotide polymorphisms in POAG pathogenesis and gender, as well as paracentral visual field defects. They confirmed significant associations between ten *CAV1/CAV2* single nucleotide polymorphisms and POAG pathogenesis. Nine of them were significant only in women and five of them were correlated with POAG with early paracentral visual field defects. Besides, none of the investigated single nucleotide polymorphisms were associated with POAG with peripheral visual field loss only or POAG among men. Thus, the above-mentioned data confirmed a role of *CAV1, CAV2*, or both of them in POAG, and suggested that the caveolins may affect POAG pathogenesis in women and in patients with early paracentral -visual field defects [224]. Another study by Wiggs et al. has reported that the single nucleotide polymorphisms are associated with POAG pathogenesis in American Caucasian population. In the same research, it was also confirmed that associations with several *CAV1/CAV2* single nucleotide polymorphisms, such as rs1052990 and rs4236601, are significant mostly among women [103].

#### 3.2.3. *SIX1/SIX6*

Both *SIX1* and *SIX6* are located on 14q22.3-q23.3 chromosome [225]. Members of the *SIX* family of homeoproteins are expressed in various tissues during vertebrate embryogenesis, and constitute a crucial regulators of the cell development, proliferation, differentiation, survival, and migration. In vivo studies have shown that the *SIX* family members are important both during organogenesis and tissue specification [226,227,228,229,230].

*SIX1* is commonly expressed in otic vesicles, nasal pits, branchial arches, and in dorsal root ganglia and somites, which give rise to the skeletal muscle of the trunk and limbs [231]. It has been reported that *SIX*1 plays a key role in the development of the mammalian retina [232]. *SIX6*, also known as *Optx2*, is expressed in the ventral optic stalk, which constitutes a structure that precedes the optic nerves embryologically. It has been reported that *SIX*6 is associated with congenital glaucoma pathogenesis. There is ample evidence that it is also correlated with anophthalmia in both mice and humans. Moreover, *SIX*6 is directly involved in the eye development [233,234]. It has been reported that *SIX6* is expressed in the ganglion cell layer and inner nuclear layer, as well as in the developing and adult human retina, optic nerve, and other brain structures such as hypothalamic and pituitary regions [235,236].

The significance of the *SIX1/SIX6* locus in glaucoma has been previously discovered for VCDR and POAG, whereas the subsequent research confirmed a direct correlation between polymorphisms in this region and glaucoma onset [237]. A significant correlation between single nucleotide polymorphism rs10483727 located in *SIX1/SIX6* and POAG pathogenesis has been reported [101,238]. Furthermore, it has been demonstrated that rs10483727 is associated with VCDR, which constitutes an important optic nerve parameter clinically used to diagnose and monitor POAG progression [205]. Carnes et al. have sequenced the *SIX6* coding and regulatory regions in 262 POAG cases and 256 controls and identified six nonsynonymous coding variants, namely five rare and one common variant, Asn141His (rs33912345), that has been strictly correlated with POAG pathogenesis in the NEIGHBOR/GLAUGEN datasets. It has been demonstrated that homozygous for the *SIX6* risk allele (His141) individuals have a statistically thinner retinal nerve fiber layer as compared with homozygous for the *SIX6* non-risk allele (Asn141) individuals. The results obtained by Carnes et al. have led to the conclusion that *SIX6* risk variants disrupt the development of the neural retina, resulting in a reduced number of RGCs, and hence increased risk of glaucoma-associated loss of vision [234]. Kou et al. have reported that the T risk allele of the lead single nucleotide polymorphism, rs10483727, localized in *SIX1/SIX6* was directly connected with a decrease in the global and different sectoral retinal nerve fiber layer (RNFL) thickness in individuals of European descent. Individuals with more copies of the risk allele exhibited a significantly thinner RNFL. Besides, individuals with the heterozygous genotype have also been found to have an intermediate level of RNFL thickness as compared with the homozygous groups [239]. Cheng et al. have evaluated the association between the *SIX6* missense variant rs33912345 and RNFL thickness by spectral-domain optical coherence tomography in the Singapore Chinese subjects. It has been demonstrated that non-glaucomatous subjects with the *SIX6* missense variant exhibited reduced RNFL thickness in regions mainly affected by glaucoma. Thereby, it could be concluded that it may constitute the major mechanism for increased risk of POAG in individuals with the *SIX6* His141 risk variant [240]. The results obtained by Sang et al. have suggested that two single nucleotide polymorphisms at the *SIX1-SIX6* locus, namely rs10483727 and rs33912345, are significantly correlated with HTG, NTG, and overall POAG, especially with an increased incidence risk of NTG in the Chinese population. Besides, it has been demonstrated that the correlation between rs10483727 and rs33912345 variants and POAG pathogenesis was significant in patients aged between 20 and 40 years, but not in those aged above 40 years in the HTG group, whereas in the NTG individuals, the genetic association has been found in both younger and older subgroups for rs33912345. For rs10483727, a direct correlation has been indicated only for individuals with NTG above 40 years old [241]. A significant association between rs10483727 (C > T) variant in *SIX1/SIX6* locus and POAG pathogenesis has also been confirmed in the Saudi Arabia population [242].

Shah et al. have not established a significant correlation between the rs10483727 and rs33912345: c.421A > C variants and PAOG pathogenesis in the South Indian population, whereas subjects carrying the corresponding C or T risk alleles exhibited a dose-dependent reduction in the thickness of the retinal nerve fiber layer and a significant increase in the VCDR. Shah et al. have further support for the implication of *SIX6* variants in the POAG pathogenesis, as well as the *SIX*6 haploinsufficiency. This study also demonstrated that the newly identified 4 bp deletion significantly reduced reporter expression in RGCs and amacrine layers, where human *SIX6* is expressed [243]. Moreover, Mohanty et al. have reported that *SIX6* plays a crucial role in POAG pathogenesis, as two novel mutations p.R116G and p.R116E in the *SIX6* were found in North Indian POAG individuals. Replacement of R116 by G or E might evoke loss of interaction between DNA and R116 of wild type (WT) *SIX*6 protein. Individuals with the p.R116E mutation exhibited not only significantly more visual field damage, but also earlier age of onset of the disease [244].

#### 3.2.4. *TMCO1*

*TMCO1*, also known as *HP10122*, is ubiquitously expressed in multiple developing and adult human tissues, including the ocular tissues. *TMCO1* encodes a transmembrane protein with a coiled-coil domain that may localize to the Golgi apparatus and ER or to the mitochondria within different cell types. It has been reported that the protein sequence is completely conserved among many mammalian species. *TMCO1* has been identified in retinal cells, whereas the strongest expression has been reported in RGCs. Moreover, it has been demonstrated that TMCO1 plays a significant role in apoptotic cell death. Thus, the above-mentioned data may suggest a direct correlation of *TMCO1* with glaucoma pathogenesis, which is characterized by excessive RGCs’ apoptosis [100,245,246,247].

The physiological function of TMCO1 is not fully elucidated, whereas it has been reported that it plays a key role in the maintenance of calcium ion homeostasis within the ER [248,249]. Furthermore, it has been suggested that TMCO1 may constitute an important protein in tumor suppression as well as play a crucial role in cell cycle regulation within the ocular tissues [250,251].

Burdon et al. have identified loci rs4656461[G] near *TMCO1* on chromosome 1q24 associated with severe POAG-mediated visual field loss in a GWAS of a Caucasian cohort [100]. The same *TMCO1* rs4656461 variant was correlated with POAG pathogenesis in the Pakistani population [252]. Moreover, rs4656461 and rs7555523 variants at *TMCO1* showed significant association with POAG in the Chinese population, as carriers of these risk alleles at *TMCO1* seemed to be predisposed to the development of high-tension POAG [253]. However, a study by Kondkar et al. has reported that rs7555523 variant in *TMCO1* as well as related clinical indices including IOP and CDR are not correlated with POAG pathogenesis in the Saudi Arabian cohort [254]. Research by Sharma et al. has demonstrated a direct correlation between genetic variations both in and around *TMCO1* with age at the diagnosis of POAG. Outcomes obtained in this study have suggested that individuals homozygous for the rs4656461 risk allele (GG) are 4–5 years younger at the date of diagnosis than noncarriers of this allele. Moreover, it has been shown in this study that *TMCO1* is expressed in most tissues in the human eye, including the TM and retina. The cytoplasmic and nuclear inclusions of endogenous TMCO1 in the human ocular tissues have been confirmed [251]. Koolwijk et al. have reported that IOP, a highly heritable risk factor for POAG, is significantly associated with rs7555523 located in *TMCO1*. Moreover, *TMCO1* has been confirmed to be highly expressed in the ciliary body, TM, lamina cribrosa, optic nerve, and retina. Interestingly, it has also been shown that *TMCO1* functionally interacts with other glaucoma-associated genes including CAV1 [106]. Besides, Verkuil et al. have demonstrated a direct association of an another single nucleotide polymorphism, namely rs4657473 (C > T), in *TMCO1* with POAG in African Americans population [255].

#### 3.2.5. *GAS7*

The *GAS7* belongs to the Pombe Cdc 15 homology (PCH) family [256]. It has been reported that growth arrest-specific (GAS) proteins play an important role in the regulation of multiple biological processes such as microfilament organization, neuronal differentiation, apoptosis, tyrosine kinase receptor activity, and control of the cell cycle progression [257,258,259,260,261,262]. *GAS7*, located on chromosome 17p13.1 [106], is expressed in early embryonic cells, testis, and neurons of several regions of the brain [263,264,265,266]. Furthermore, it has been demonstrated that *GAS7* is expressed in the optic nerve and lamina cribrosa, which belongs to the connective tissue network via which the nerve fibers traverse to create the optic nerve, and it is predicted that the mentioned structure may constitute the main site for glaucomatous damage to the optic nerve. A moderate to high expression of *GAS7* has also been demonstrated in the ciliary body, which produces the aqueous humor, and high expression of *GAS7* has been found in the TM, which is the major tissue involved in aqueous humor outflow. Both ciliary body and TM are responsible for IOP level controlling [106]. Moreover, high expression of *GAS7* has been demonstrated in amacrine cells in the mouse retina, whereas lower expression *GAS7* has been demonstrated in retinal cell types, which are usually not affected by glaucoma [267]. *GAS7* may interact with other genes implicated in glaucoma pathogenesis such as *MYOC*, *OPTN*, *WDR36*, *CAV1*, *nitric oxide synthase 2* (*NOS2)*, *forkhead box C1* (*FOXC1)*, *apolipoprotein E* (*APOE)*, *amyloid precursor protein* (*APP),* and *clusterin* (*CLU)* [106]. It has been reported that *GAS7* interacts with *MYOC* and *CAV1* via β-catenin (CTNNB1) and RhoA (RHOA). β-catenin constitutes a part of the Wnt signaling pathway, which is implicated in trabecular outflow regulation, whereas RhoA signaling is responsible for regulation of the intracellular levels of phosphorylated myosin light chain, which directly influences TM cellular contraction and aqueous humor outflow [268,269]. It has also been reported that *GAS7* is regulated by transforming growth factor (TGF) beta, which is implicated in trabecular outflow and the optic disc development [270].

Investigation by Koolwijk et al. has identified the rs11656696 polymorphism located in *GAS7* and demonstrated that it is associated with IOP level in subjects from four independent population-based studies from the Netherlands, as well as from four additional cohorts from the United Kingdom, Australia, Canada, and the Wellcome Trust Case-Control Consortium 2/Blue Mountains Eye Study. The analysis has demonstrated that the rs11656696 polymorphism is directly linked with glaucoma pathogenesis. Interestingly, in subjects from four additional cohorts, each copy of the rs11656696[A] minor allele was correlated with a 0.19 mmHg decrease in IOP [106]. A recent study by Xu et al. has demonstrated that rs11656696 polymorphism in *GAS7* is directly correlated with POAG pathogenesis and may constitute a protective factor against POAG in a Chinese population. The minor [A] allele frequency of rs1165669 polymorphism was 0.477 in the POAG cases, whereas it was 0.526 in controls. It has been reported that individuals carrying rs11656696 AA genotype were less likely to suffer from POAG than individuals carrying AC/CC genotypes [271]. However, Kondkar et al. have reported that polymorphism rs11656696 is not associated with IOP and CDR, thereby it is not considered a risk factor for POAG in the Saudi Arabian cohort [272].

#### 3.2.6. *ATOH7*

*ATOH7*, also known as *Math5*, is located on 10q21.3-22.1 chromosome. It has been reported that ATOH7 is a single exon gene that encodes a basic helix-loop-helix (bHLH) transcription factor. There is ample evidence that bHLH transcription factors are responsible for retinal nerve formation in the vertebrates as well as in the invertebrates. Brown et al. have demonstrated that human *ATOH7* plays a crucial role both in the RGCs and optic nerve formation. Thereby, it has been suggested that mutations in *ATOH7* may trigger a congenital malformations or degenerative diseases of the optic nerve [273]. Besides, using an in vivo experimental model, it has been confirmed that *ATOH7* transcription factor catalyzes the rate-limiting step in the specification of RGCs [274]. Furthermore, it has been reported that *ATOH7* constitutes an important protein in the differentiation of Müller cells-derived retinal stem cells into RGCs in a rat model of glaucoma [275]. There is a lot of other evidence that *ATOH7* plays a key role in formation of RGCs and optic nerve. It has been demonstrated that *ATOH7*^-/-^ mice showed deprivation of RGCs and optic nerve formation. Moreover, lack of *ATOH7* also resulted in thinner retinas, fewer rod bipolar cells, Muller glia, and calretinin-positive amacrine cells, but relatively more cones and cholinergic amacrines, as compared with wild-type mice [276,277,278]. Moreover, a recent study by Zhang et al. has confirmed that human *ATOH7* possesses a high potential in promoting early retinogenesis and specifying the RGC differentiation program, hence it provides insight for manipulating RGCs formation from stem cell-derived retinal organoids [279]. However, it has been demonstrated that expression of *ATOH7* alone is insufficient for direct differentiation of RGCs during normal retinal development. *ATOH7*-expressing cells give rise to multiple retinal cell types such as RGCs, amacrine, horizontal, and photoreceptor cells. It has been suggested that *ATOH7* plays a crucial role in determining the RGC competence of retinal progenitors and is also responsible for the activation of key transcription factors in RGCs’ development [280].

Multiple GWAS have reported a strong correlation between VCDR, commonly used to identify and monitor glaucomatous damage to the optic nerve, and rs7916697 polymorphism near *ATOH7* in two Australian twin cohorts, the Rotterdam study cohorts, and a Latino population [205,281,282]. Additionally, a suggestive protective association has been reported between the rs7916697 polymorphism in *ATOH7* and POAG in the Afro-Caribbean population of Barbados [203].

GWAS have also reported a link between *ATOH7* and *raftlin lipid raft linker 1* (*RFTN1*) and glaucoma-related optic disc parameters. *ATOH7* and *RFTN1* polymorphisms have been demonstrated in POAG individuals and their relationships with VCDR and CCT have been confirmed. Chen et al. have demonstrated that combination of *ATOH7* (rs3858145 GG) and *RFTN1* (rs690037 TT) polymorphisms may significantly increase risk of POAG development [283].

Fan at al. have revealed that POAG risk, which is associated with increased VCDR, was significantly influenced by the C allele of rs1900004 polymorphism in *ATOH7* in an American Caucasian population [238]. Furthermore, the rs1900004 polymorphism in *ATOH7* has also been reported as a non-IOP-related genetic risk factor for NTG in a Japanese population [284]. Philomenadin et al. have suggested that rs1900004 polymorphism in *ATOH7* may constitute a risk factor for POAG development only upon interaction with variants of other candidate genes in an Indian population [107]. However, a link between rs1900004 polymorphism in *ATOH7* and POAG risk or its related clinical indices such as IOP and CDR has not been confirmed in a Saudi Arabian cohort [285].

#### 3.2.7. *RPGRIP1*

The human *RPGRIP1* gene is located on chromosome 14q11 and is expressed as multiple splice variants [286]. *RPGRIP1* consists of *N*-terminal and coiled-coil domains followed by a C2 domain and a C-terminal RPGR-interacting domain (RID). There is ample evidence that *RPGRIP1* is highly expressed in the human retina [287,288,289]. It has been suggested that *RPGRIP1* expression is enriched in retinal photoreceptors, where it is stably associated with the connecting cilia [290,291]. Furthermore, it has been demonstrated that *RPGRIP1* is also strongly expressed in a subset of inner retinal neurons, namely in the amacrine cells [292]. The function of *RPGRIP1* has not yet been fully elucidated [293,294]. Interestingly, *RPGRIP1* has been identified in the retina as a complex with the CEP290 protein as well as in the amacrine cells with the neuronal nucleoporin RANBP2 [295]. Thereby, it is predicted that *RPGRIP1* isoforms may constitute a plastic and dynamic scaffold for proteins or protein modules of specific signaling pathways of different retinal cell subpopulations [296,297]. Besides, it has been suggested that *RPGRIP1* may participate in ciliary protein transport [294]. Moreover, it has been suggested that *RPGRIP1* is directly implicated in various forms of glaucoma, including POAG. Fernandez-Martines et al. have demonstrated that heterozygous non-synonymous *RPGRIP1* variants may cause or increase the susceptibility to glaucoma, as well as that disrupted interaction of *RPGRIP1* with other proteins may result in glaucoma development in European individuals [105].

## 4. Endoplasmic Reticulum Stress and the Unfolded Protein Response Signaling Pathway

The newest data have reported that aggregation of misfolded and unfolded proteins within the lumen of the ER is strictly associated with the pathogenesis of multiple neurodegenerative disorders, as it may affect numerous cell signaling pathways and neuronal connectivity, and finally evoke neuronal apoptosis [298,299,300,301]. The ER constitutes a dynamic cellular organelle that plays a major role in protein synthesis, posttranslational modification, trafficking, lipids and steroids synthesis, carbohydrate metabolism, calcium homeostasis, as well as efficient drugs metabolism [302,303,304]. ER homeostasis is maintained via ER chaperones including glucose-regulated protein 78 (GRP78), also known as immunoglobulin heavy chain-binding protein (BiP), that promote proper proteins folding into functional proteins, maintain proteins in a folded state, prevent aggregation of protein folding intermediates, and direct aberrant proteins to ER-associated protein degradation (ERAD) [305,306]. Multiple factors, including significantly increased protein translation, oxidative and osmotic stress, depletion of energy and ER calcium level, acidosis, drug-induced toxicity, gene mutations, viral infection, as well as increased temperature, may markedly enhance the rates of aberrant proteins within the ER [307,308,309,310,311]. Disruption of the ER homeostasis directly triggers accumulation of misfolded and unfolded proteins within the ER lumen, and subsequently evokes activation of the unfolded protein response (UPR) signaling branches to decrease the level of aberrant proteins within the ER lumen and thereby restore homeostasis [312]. However, if the ER stress is severe and prolonged, the pro-adaptive branch of the UPR may switch into the pro-apoptotic one. Thus, there is ample evidence that targeting components of the UPR signaling pathway may constitute a novel, promising treatment strategy against ER stress-dependent human neurodegenerative pathologies [301,313,314].

Protein kinase RNA-like endoplasmic reticulum kinase (PERK), inositol requiring enzyme-1 (IRE1), and activating transcription factor 6 (ATF6) constitute the three major transducers of the UPR signaling pathway [315,316,317,318]. It has been reported that PERK, a serine/threonine ER kinase, is firstly activated among all of the three branches of the UPR signaling pathway [319,320,321,322]. PERK belongs to the eIF2α kinase subfamily and is composed of a luminal domain and cytoplasmic domain with serine/threonine protein kinase activity [323,324]. Under physiological conditions, all three UPR receptors are maintained in an inactive state by GRP78 chaperones, whereas an increased level of aberrant proteins within the ER lumen promotes dissociation of the GRP78 proteins from the UPR effectors, which directly evokes activation of the mentioned transducers [325,326]. In turn, PERK undergoes oligomerization and autophosphorylation, which subsequently activate its kinase domain so as to induce the UPR signaling pathway cascade [327]. The main downstream target of PERK constitutes eukaryotic initiation factor 2α (eIF2α), and becomes phosphorylated under ER stress conditions, resulting in attenuation of global protein translation and, on the other hand, enhanced translation of only selective proteins including activating transcription factor 4 (ATF4). ATF4 plays a dual role, because, as a transcription factor, it may increase the expression of proteins responsible for cells adaptation to mild or moderate ER stress conditions, or, under severe and chronic ER stress, it promotes expression of pro-apoptotic proteins, including CCAAT-enhancer-binding protein homologous protein (CHOP) [328,329]. It has been reported that CHOP-mediated apoptotic cell death is strictly correlated with the enhanced expression of multiple pro-apoptotic genes such as B-cell lymphoma-2 (BCL-2), growth arrest and DNA damage-inducible protein (GADD34), endoplasmic reticulum oxidoreductin 1α (ERO1α), or tribbles-related protein 3 (TRB3) [330,331,332].

## 5. The Role of the ER Stress-Dependent Unfolded Protein Response Signaling Pathway in POAG Pathogenesis

**Figure 3:**
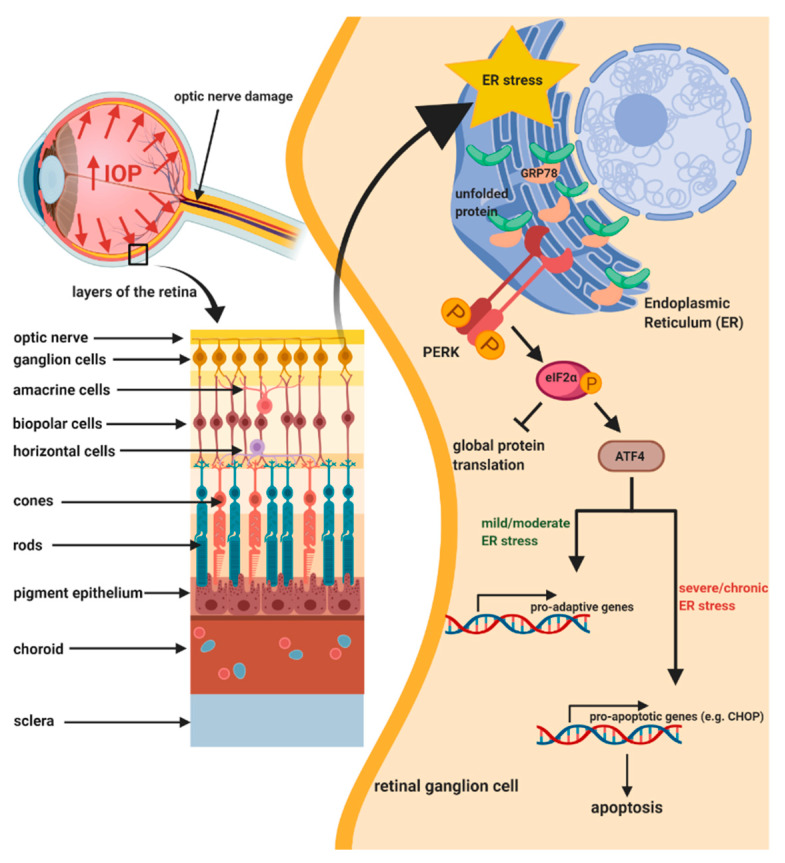
Activation of the protein kinase RNA-like endoplasmic reticulum kinase (PERK)-dependent UPR signaling pathway within retinal ganglion cells (RGCs). POAG pathogenesis, on the molecular level, is correlated with the accumulation of aberrant proteins, such as mutant myocilin, within the ER lumen, which evokes ER stress conditions within the RGCs, subsequent significant elevation of the intraocular pressure (IOP) and activation of the PERK-dependent unfolded protein response (UPR) signaling pathway. Under mild to moderate ER stress conditions, UPR has a pro-adaptive role, whereas severe or long-termed ER stress conditions trigger activation of the pro-apoptotic branch of the UPR, directly leading to RGCs’ apoptosis.

ER stress may be evoked by overexpression of genes or gene mutations, which results in protein aggregation as well as other molecular processes, the major role of which is to prevent the nascent protein from the processing via the ER. It has been demonstrated that genetic abnormalities that directly disrupt the proper ER function may subsequently induce UPR signaling pathway, resulting in cell death (Figure 3). There is ample evidence that ER-mediated apoptosis may contribute to the development and progression of several diseases including ocular diseases [91]. Interestingly, there is increasing evidence confirming that ER stress and UPR signaling pathway are directly implicated in POAG pathogenesis, as one of the neurodegenerative, ocular diseases [114,333,334,335].

Mutation in *MYOC* constitutes one of the major causes of POAG development at the genetic level. *MYOC* missense mutations may be strictly correlated with activation of the ER stress-mediated UPR signaling pathway [336,337,338]. Zhou et al. have demonstrated that mutant myocilin is characterized by lower solubility than the physiological form of myocilin. Thus, one of the major causes of the ER stress induction by the mutant myocilin may be associated with its higher ability to aggregation, as compared with the normal form of myocilin [109]. Moreover, another study by Aroca-Aguilar et al. has shown that disease-causing *MYOC* mutations significantly reduced myocilin solubility, which directly promoted its aggregation within the ER lumen [339]. The pathological mechanisms responsible for outflow resistance within the TM as well as subsequent elevation of the IOP have not yet been fully elucidated. Recent studies have demonstrated that accumulation of the unfolded or misfolded proteins within the ER lumen may directly induce ER stress conditions, resulting in significant elevation of the IOP as well as TM damage [340]. It has been demonstrated that disrupted ability of the UPR to remove aberrant mutant or damaged proteins such as myocilin may trigger ER stress, which subsequently leads to impairment of TM cells [114,335]. Accumulated mutated myocilin within the ER lumen evoked overexpression of GRP78 and protein disulfide isomerase. Besides, mentioned molecular events resulted in deformed cellular morphology and diminished cell proliferation, which constituted a major cause of TM cells’ dysfunction, which may play a key role in glaucoma pathogenesis [114]. Topical ocular sodium 4-phenylbutyrate (PBA) treatment rescued glaucoma phenotypes in a transgenic mouse model of POAG caused by the Y437H *MYOC* mutation *(Tg-MYOC^Y437H^)*. Topical PBA markedly improved secretion of myocilin, and reduced its aggregation and subsequent ER stress conditions in the TM of *Tg-MYOC^Y437H^* mice. Furthermore, it has also been demonstrated that topical PBA evoked significant reduction of ER stress-mediated IOP level in WT mice [338]. Furthermore, it has been found that, in Tg-*MYOC*^Y437H^ mutant, myocilin aggregated in the lumen of the ER in the TM, resulting in the induction of ER stress conditions. Additionally, severe and long-term ER stress conditions have been closely associated with increased IOP and TM apoptotic cell death in an in vivo model of Tg-*MYOC*^Y437H^ mice. Interestingly, phenylbutyric acid-mediated reduction of ER stress promoted secretion of mutant myocilin in the aqueous humor and significantly declined its deposition within the ER, resulting in the prevention of TM apoptotic cell death in Tg-*MYOC*^Y437H^ mice [336].

It has been reported that the ER stress-dependent UPR signaling pathway activation is closely correlated with glaucoma pathogenesis. After induction of ER stress conditions via treatment of RGCs with tunicamycin, the levels of GRP78, phosphorylated form of eIF2α (p-eIF2α), and CHOP were significantly increased. Hence, the mentioned data indicate that apoptosis of RGCs may occur in the ER stress-dependent manner. Interestingly, it has also been reported that the levels of BiP and CHOP, as one of major markers of the UPR signaling pathway activation, were significantly increased in retinal cells after *N*-methyl-D-aspartate (NMDA)-induced injury. Thereby, the above-mentioned molecular event may confirm a correlation between ER stress and glaucoma pathogenesis [341]. Moreover, a study by Doh et al. has also demonstrated that ER stress and PERK-dependent UPR signaling pathway play a critical role in the RGCs’ apoptotic cell death. In the mentioned study, the levels of GRP78, p-PERK, and p-eIF2α, induced by an elevated IOP, were markedly increased during the early stage of the UPR signaling pathway activation to protect RGCs against apoptosis. On the other hand, in the case when the increased IOP was prolonged, the CHOP expression was also significantly increased, directly leading to apoptotic cell death of RGCs at the late stage of the UPR signaling pathway activation. Thus, the above-mentioned data confirm a strong correlation between ER stress-mediated PERK/p-eIF2α/CHOP signaling pathway activation and RGCs’ cell death in chronic glaucoma [342].

Furthermore, a study by Zode et al. has demonstrated that chronic ER stress plays a critical role in OHT development in an in vivo mouse model of glaucoma induced by glucocorticoid. Treatment of WT mice with dexamethasone evoked an elevation of IOP, loss of RGCs, as well as axonal degeneration. Interestingly, increased IOP has been associated with persistent ER stress of the TM. Besides, an elevated expression of CHOP, a major marker of the ER stress-mediated apoptosis, has been found in the anterior segment tissues. Deletion of *CHOP* suppressed ER stress in mentioned tissues and also prevented dexamethasone-mediated OHT [333]. Moreover, a recent study by Wang et al. has confirmed a link between ER stress-mediated activation of the UPR signaling pathway and glaucoma pathogenesis. Both TM stem cells and TM cells were treated with the ER stress inducers, whereas a significantly elevated expression of ER stress markers, such as GRP78 and CHOP, was demonstrated only in TM cells in comparison with TM stem cells [343]. Moreover, a study by Peters et al. has confirmed that inability of TM cells to suppress ER stress evokes enhanced expression of CHOP, which may evoke not only IOP elevation, but also loss of TM cells in the apoptotic-dependent manner. It has been demonstrated that persistent ER stress plays a crucial role in glaucoma development, because the levels of ER stress markers including GRP78, GRP94, ATF4, ERO1α, and CHOP were markedly increased in glaucomatous TM cells as compared with normal TM cells [334]. A study by Yang et al. has demonstrated that components of the ER stress-mediated UPR signaling pathways may constitute a novel therapeutic targets for glaucoma as well as other neurodegenerative disorders. The above-mentioned study showed that opposite manipulation of the UPR signaling pathway, namely inhibition of PERK/eIF2α/CHOP branch of the UPR and activation of the X-box-binding protein 1 (XBP1), results in RGCs axons and somata survival and preserves visual function [344].

Ultimately, the above-mentioned data have confirmed a hypothesis that the induction of PERK-dependent UPR signaling pathway under ER stress conditions may be closely correlated with POAG pathogenesis at the molecular level. Hence, targeting of the components of the UPR signaling branches may contribute to the development of a novel, ground-breaking treatment strategy against POAG.

## 6. Summary and Perspective

POAG, the most common form of the glaucoma, rarely causes symptoms until it is at the advanced stage, thus it is commonly known as a ‘silent thief of sight’. The currently existing approach for treatment against POAG is primarily limited to the reduction of the IOP and may evoke numerous side-effects in POAG patients. Furthermore, it does not take into account the molecular processes occurring in the ocular tissues. As proven above, there are multiple glaucoma-associated genes, whereas their detailed functions in the disease pathogenesis and progression are not fully elucidated. Thereby, a precise characterization of genes that are directly linked to POAG pathogenesis constitutes a crucial step to develop gene-based diagnostic tests, which will detect disease earlier and predict response to drugs and treatment, and it may also contribute to the development of novel gene therapies. Moreover, the newest data have demonstrated that ER stress and PERK-dependent UPR signaling pathway may play a crucial role in POAG pathogenesis at the molecular level. Thus, components of the PERK-mediated UPR signaling pathway, implicated in POAG pathogenesis, may constitute a potential targets of a novel, ground-breaking treatment approach against POAG, which could prevent the disease development in the future.
